# Post-traumatic stress disorder and depression among Syrian refugees residing in the Kurdistan region of Iraq

**DOI:** 10.1186/s13031-019-0238-5

**Published:** 2019-11-08

**Authors:** Harem Nareeman Mahmood, Hawkar Ibrahim, Katharina Goessmann, Azad Ali Ismail, Frank Neuner

**Affiliations:** 1 Department of Psychology, Clinical Psychology and Psychotherapy, Bielefeld University, Bielefeld, Germany; 2 vivo international, Konstanz, Germany; 3 Department of Clinical Psychology, Koya University, Koya, Kurdistan Region of Iraq, Iraq

**Keywords:** Refugees, Syria, War, PTSD, Depression

## Abstract

**Background:**

Since the Syrian civil war began in March 2011, more than half of the Syrian population was forced to escape from their homes, and more than 5 million of them fled their country. The aim of the present study is to estimate the psychological consequences of this conflict among the refugee population who fled to Iraq.

**Method:**

In 2017, a team of locally trained psychologists and social workers interviewed 494 married couples (988 individuals) who were Syrian Kurdish refugees in the Kurdistan Region of Iraq. Validated Kurdish Kurmanji and Arabic versions of post-traumatic stress disorder (PTSD) Checklist for DSM-5 and depression section of Hopkins Symptom Checklist-25 were used for assessing PTSD and depression symptoms.

**Results:**

Almost all of the participants (98.5%) had experienced at least one traumatic event and 86.3% of them experienced three or more traumatic event types. The prevalence of probable PTSD was about 60%. Gender, length of time in the camp, area in which participants were grown up, and the number of traumatic event types were significant predictors for the presence of PTSD symptoms. Approximately the same rate of participants (59.4%) experienced probable depression, which was associated with gender, age, time spent in the camp, and the number of traumatic event types.

**Conclusion:**

PTSD and depression are prevalent among refugees exposed to traumatic events, and various variables play important roles. The pattern of risk factors in this population is consistent with findings from war-affected populations in other regions and should be considered for intervention within this population and more broadly.

## Introduction

Over recent years, the number of refugees has increased tremendously worldwide [1]. The main reasons fueling this global refugee crisis and mass migration were conflict, war, persecution, violation of human rights, and economic and political crises [2, 3]. By the first half of 2017, the number of refugees worldwide increased to over 18.5 million, more than half of whom fled from Syria, South Sudan, and Afghanistan. This number is considered to be the largest number of refugees and worst humanitarian crisis since the Second World War [4]. Since the Syrian conflict has escalated in March 2011, hundreds of thousands of Syrian civilians have been injured, killed, and kidnapped. This has led to a massive forced displacement as a result of the multi-state conflict occurring in Syria [5, 6]. By the end of 2017, 5.4 million Syrian refugees were registered in the neighboring countries of Turkey, Jordan, Egypt, and Iraq [7]. As reported by the United Nations High Commissioner for Refugees (UNHCR) in March 2018, the number of Syrian refugees in Iraq had reached about 250,000, almost all of them residing either in camps or in urban areas in the Kurdistan Region of Iraq (KRI) [8].

As a highly vulnerable population, refugees in particular have often been exposed to traumatic events such as torture, rape, murder and even genocide in their home countries [2, 9]. In addition to war-related violent events experienced in their country of origin, refugees are exposed to danger and potentially traumatic events during the course of their flight [9, 10]. Therefore, when they arrive at camps or host countries, many already suffer from psychological and physical impairment. These potentially traumatic events and impaired quality of life are associated with different types of psychological disorders prevalent among refugees [10]. Studies have also confirmed that the prevalence of psychological disorders is relatively high among conflict affected populations [11], and that the effect and symptoms of traumatic experiences may last for years [12–15]. In sum, psychological illness is common among already highly vulnerable populations and it can persist over time if there is no adequate treatment or intervention.

In most epidemiological surveys and studies on psychopathology of war survivors, PTSD and depression are among the most prevalent mental health issues [16–21]. An umbrella review by Turrini and colleagues which summarized thirteen systematic review studies on the prevalence of common mental disorders among asylum seekers and/or refugees found that PTSD and depression were two of the most frequent mental disorders experienced by those populations with prevalence rates at 30–40% [3]. Similar figures were obtained in meta-analyses [19, 22]. It is likely that negative mental health consequences are more common among refugees as a result of war and living difficulties after migration [23]. However, across studies, prevalence rates of PTSD and other mental health problems among war-affected populations vary widely. For example, based on a meta-analysis conducted in 2015, 5 and 9% of adult refugees who were living in Western countries were diagnosed with major depression and PTSD, respectively [17]. In single studies, rates of PTSD and depression range between 4.4 and 86% for PTSD and 2.3 and 80% for depression [24]. Moreover, a study among a population affected by armed conflict in Eastern Afghanistan indicated that 20.4 and 38.5% of the participants met the criteria for PTSD and depression symptoms, respectively [25]. There may be several explanations for these differences in reported prevalence, such as the length of time between the exposure to potentially traumatic events and the assessment process, or as a result of the severity and number of events that participants have experienced [26]. In addition, methodological factors, in particular the use of cross-culturally validated vs. non-validated instruments, play a major role in the explanation of these differences [22, 24].

This variance in prevalence rates is also reflected in studies of Syrian refugees. For example, based on a recent study by Acarturk and colleagues that used the Impact of Event Scale–Revised for PTSD and the Beck Depression Inventory for depression among Syrian refugees residing in Turkey, the prevalence of PTSD and depression were 83.4 and 37.4%, respectively [9]. Using the Harvard Trauma Questionnaire among Syrian Kurdish refugees living in the KRI, Ibrahim and Hassan found that 35–38% of participants met the criteria for a PTSD diagnosis [27]. Further, based on two other studies using an Arabic version of the Mini International Neuropsychiatric Interview among Syrian refugees living in camps in Lebanon, the prevalence of PTSD and depression were 27.2 and 43.9%, respectively [28, 29]. So, the different prevalence rates found in previous studies seem to be related, at least in part, to the variety of diagnostic instruments used and the absence of adapted tools to measure PTSD and depression among Syrian refugees.

The contribution of assessment factors was further illustrated by studies with Kosovar refugees, with two studies finding prevalence rates of PTSD of 60.5% based on instruments that had not been validated for this population [30, 31]. However, when using psychiatric interviews among the same population, only 23.5% of them were diagnosed with PTSD [32]. When conducting work in such populations, it is critical to utilize instruments that were validated for the specific population since, although there are shared symptoms of PTSD among cultures, the meanings and understanding for normality of the symptoms might differ [2]. For example, positive symptoms of PTSD like hypervigilance and intrusive thoughts seem to be more severe among Hispanic adults than African Americans [33]. Thus, one common limitation among previous studies of non-Western refugee populations is the lack of validated instruments to assess mental health problems that reflect the populations’ culture, political background, and economic and educational status, among other characteristics [26, 34, 35].

Being female is often one of the identified risk factors that influence the prevalence of mental disorders among refugees and displaced people. A recent meta-analysis in war-affected populations indicated that studies with higher percentages of women reported higher prevalence rates of PTSD [20]. Studies also showed that the prevalence of rates these disorders were often twice as high among women compared to men [9, 25, 30, 36–38]. Moreover, Ekblad and colleagues argued that being female is one of the risk factors for developing mental health problems as women are more exposed to rape and other forms of gender-based violence, and are at a greater risk of losing their spouse [39]. However, a study in Lebanon reported no significant difference in the presence of PTSD and depression between male and female university students in Beirut who experienced war-related events [40]. More broadly, the lifetime prevalence and duration of PTSD in the general population is greater among women than among men [41].

Similar to gender, age seems to be associated with the presence of symptoms of mental disorders in the wake of war-related trauma [42]. Most of the studies with higher mean age in war-affected populations reported higher levels of depression [20]. With respect to Syrian refugees, a recent study among Syrian refugees resettled in Sweden found that mental disorders, including PTSD and depression, were more common among older refugees [38]. It has been also confirmed that older age (adults aged 65 or older) is a risk factor in the development of mood disorders, including depression [43].

Next to socio-demographic characteristics, higher numbers of experienced traumatic events significantly predict the development of mental disorders among refugees. As a result of negative events experienced prior to migration, refugees may have increased vulnerability to developing mental disorders compared to the general population [44]. Studies among populations exposed to mass conflict and displacement have reported that the number of potential traumatic events plays a significant role in the increase of rates of mental disorders [22]. In this regard, studies among Syrian refugees also showed that a higher number of traumatic events is a significant factor that predicts mental health problems [36, 45].

In addition, geographical factors seem to have an impact on mental health in refugee populations. Across studies, the prevalence of mental disorders among refugees is related to both the country of origin as well as the country of resettlement [24]. A study of former Yugoslavians who fled to Germany, Italy, or the UK revealed different prevalence rates of mental disorders across countries that could only partly be explained by socio-demographic characteristics, post-migration factors, and war-trauma severity [23]. Likewise, among Syrian refugees, Cheung Chung and colleagues found that Syrian refugees residing in Turkey reported a higher level of PTSD, psychiatric co-morbidity, and trauma characteristics compared to refugees of the same origin residing in Sweden [46]. In general, results of epidemiological studies showed that the prevalence of serious mental disorders was higher in metropolitan cities in comparison to rural areas [47].

Moreover, daily stressors and living condition in the host country seems to be among factors that influence the mental health of refugees. For example, based on a model which was proposed by Miller et al., (2010), daily stressors such as living in unsafe places, lack of access to basic needs and absence of social support partially mediate the effect of war exposure in developing mental health problems [48]. In addition, according to a study among Rohingya adults residing in refugee camps in Bangladesh, daily stressors partially mediated the relationship between trauma exposure and PTSD symptoms [49].

So far, it is unclear to what extent selection factors or the difference between regional conditions cause these differences. Researchers have suggested that factors such as poor quality of living within the refugee camp [50], experiences of racism in the host country, joblessness, administration difficulties in the camp [51], and insecurity in refugees’ status and longer stay in the host country [52] contribute to the maintenance of mental health disorders and may be responsible for differences between countries.

The aim of this study was to estimate the prevalence rates of probable PTSD and depression among Syrian refugees who are living in the Kurdish region of Iraq and to determine specific risk factors they face. The majority of the Syrian population is considered to be Arab but a large minority is Kurdish (around 10%) followed by other smaller ethnic groups such as Turkmen, Assyrians, and others [52]. The majority of Kurds who fled from Syria crossed the border to the KRI.

Although formal education uses Arabic language across Syria, Kurds have their own language and culture. The study of this population is methodologically difficult, since individuals are commonly bilingual and prefer to use different languages for different occasions [53]. Some individuals would consider a mental health interview as a formal procedure and prefer to respond in Arab language, while others would prefer to reply in more colloquial Kurdish. To account for this uncertainty, the study instruments needed to be translated into both languages and implemented by bilingual interviewers according to the preferences of the respondent. We built our interview protocol upon validated instruments, including a validated and calibrated Kurdish Kurmanji and Arab version of the PTSD Checklist for DSM-5 (PCL-5) [53]. Therefore, the current study attempts to complement previous studies by using recently-validated instruments and a large sample size. It is hoped that obtaining these results will provide a clearer understanding for local and international organizations for the purpose of more effective psychological intervention among couples in this population. Finally, the findings of the present study may assist in contributing to the existing body of knowledge about mental health problems among refugees, which requires further attention.

## Method

### Participants

A total of 988 Syrian refugees (494 heterosexual married couples) took part in the current study. All of the participants were from Arbat Camp in Sulaymaniyah Governorate in the KRI. According to a published database by UNHCR in July 2017, 8479 people reside in Arbat Camp [54], and the majority of them were members of the Kurdish minority of Muslim-Sunni religion who fled from northern Syria because of military conflicts following the multi-actor conflict that began in March 2011. More than half of them (64.7%) grew up in cities and towns. The number of children in each married couple ranged from 0 to 14 (*M* = 3.45, *SD* = 2.46), 86.5% of individual participants did not have a regular income, and 51.9% of them did not have any work compared to 6% working full time. Seventy-eight percent of participants (*n* = 772) were interviewed in Kurdish Kurmanji while 216 (21.9%) of them were interviewed in Arabic. More than half of the couples (69.7%) had been married by arranged marriage by their own volition, followed by 24.2% by joint choice, 3.4% by parent’s choice, 2% by force, and .6% by other ways. See Table 1 for all socio-demographic variables.

**Table 1:** Socio-demographic information of individual participants

Variables	General	Male	Female
Age, Mean (SD)^a^	34.61 (10.96)	37.15 (10.95)	32.08 (10.38)
Marriage age, Mean (SD)^a^	22.25 (4.89)	25.1 (4.57)	20.08 (3.8)
Monthly income, Mean (SD)^b^	2563.31 (30,670.53)	3997.97 (39,397.82)	1164.6297 (18,447.87)
Number of children, Mean (SD)^c^	3.45 (2.46)	–	–
Number of male children, Mean (SD)	1.83 (1.53)	–	–
Number of female children, Mean (SD)	1.62 (1.55)	–	–
Length of stay in camp as a refugee, Mean (SD) ^a^	2.69 (1.39)	2.85(1.44)	2.54 (1.33)
Age during war, Mean (SD) ^a^	29.05 (10.89)	31.3 (10.95)	26.81 (10.36)
Number of extended family members injured during war Mean (SD) ^d^	.53 (1.61)	.59 (1.77)	.47 (1.43)
Number of extended family members killed during war, Mean (SD) ^e^	1.15 (3.38)	1.32 (4.3)	.98 (2.08)
Number of extended family members who went missing during war, Mean (SD) ^f^	.23 (.78)	.19 (.71)	.27 (.84)
Formal education, Mean (SD) ^a, g^	6.18 (4.4)	6.46 (4.09)	5.89 (4.68)
Number of lifetime displacement, Mean (SD)^h^	1.14 (.54)	1.11 (.46)	1.16 (.62)

Note: ^a^ in years. ^b^ score range: 0–600,000 IQD (1000 IQD = .74Euro). ^c^ score range:0–14. ^d^ score range: 0–20. ^e^ score range:0–62. ^f^ score range:0–8. ^g^ score range: 0–18. ^h^ score range:1–8

### Procedure

The current study was part of a larger research project which was conducted in collaboration between Bielefeld University in Germany and Koya University in the KRI. Data was collected between December 2016 and July 2017 in Arbat Camp, Sulaymaniyah Governorate. All of the participants were registered in the camp formally by the UNHCR, and they had received a shelter with some basic furniture. Since the current study was a part of a longitudinal study that was aiming for families to study partnership and family dynamics as well, so, only couples were asked to participate in the current study. So, the essential inclusion criterion to participate was the availability of both partners of the couples for interviews. The map of the camp was obtained from camps administration which was sub-divided into clusters according to approximately equal breakdowns of household and population size. The interviewers chose the tents randomly by spinning a pen from the center of the cluster, the first household that was in the straight line of the tip of the pen was selected for participation (for more details see [53, 55]). Each of the couples were interviewed in their tents by the visiting team, but in two separate rooms at the same time. During the interview, after a brief explanation about all questionnaires and answer options, the interviewers read out all the questions in each scale one by one and asked the interviewee which answer choice is suitable for them based on the scale options. The focus of this procedure was to make sure the interviewee understood the question and the answer options clearly.

Since the camp was established, psychological and psychiatric services had been provided to the population of the camp through several non-governmental organizations (NGOs). There was also a camp hospital which provided first aid psychiatric help. The first aid psychiatric unit in the camp hospital provide initial psychiatric support to people under risk (i.e., suicidal people), concerned persons will be referred to psychiatric hospitals outside of the camp if they need to be hospitalized. In addition to that, prior to the start of data collection and in cooperation with camp administration and existing NGOs, the interview team created a referral and follow-up system for those who needed psychological or psychiatric help at any point during the course of data collection. The referral pathway consists of the team director and all interviewers in cooperation with camp administration and existing NGOs in the camp. Each of the interview teams was responsible for asking visited family members for any immediate psychological services. Then, based on primary information on the case, the team director, in cooperation with camp administrations referred the case to psychological and psychiatric therapy. So, before conducting each interview, the interviewers informed the interviewees about the resources available to them for any need of their psychological and psychiatric service.

The interview team consisted of 16 clinical psychologists and social workers (8 males and 8 females). All members of the local team had at least a bachelor’s degree in clinical psychology or sociology and at least 1 year of experience working in their field. The team was divided into eight sub-teams, an in each team there were a clinical psychologist and a social worker either male or female, to conduct the interview with the participant matched by gender. Before starting the data collection, to improve skills and knowledge about the research project and necessary tasks, the interview team took part in a one-week intensive theoretical and practical training. The interviewers were taught about the questionnaires used in the study, how to conduct interviews, how to consider ethical issues, the sensitivity of culture, and mental health risk management. As part of this training, all interviewers conducted role plays as a group and as an individual under supervision. Furthermore, at the end of the training the trained team visited the camp in order to become familiar with the camp’s location, administration staff, and meet some of the NGOs who were working in the camp. All structured interviews were conducted by locally trained psychologists and social workers under supervision. The interviews with the couples took place in the couple’s tents and each interview lasted between 60 to 90 min.

As the target population is very skeptical towards any signature on any document, in particular in contexts related to their experiences and life, we refrained from obtaining signed written consent but relied on mandatory documented verbal consent (the same procedure had been used by Ibrahim and Hassan [27]).

The interviewer explained the study purposes, potential risks, and benefits based on a standardized information sheet, and then the verbal consent was documented by interviewers with their signature. The study design, procedure, consent forms, and reliance on verbal informed consents were approved by the Ethical Committee of Bielefeld University in Germany along with the Ethical Committee of Koya University in the KRI.

### Instruments

The first section of the questionnaire assessed demographic variables such as gender, age, education level, etc. This was followed by questions related to war and life (e.g., “how many people in your family were injured, killed or lost; and what was your age when this war and conflict first started; where did you live during war; and where did you grow up?”).

#### Traumatic experiences

The War and Adversity Exposure Checklist (WAEC) by Ibrahim and colleagues was employed to measure traumatic events and war-related events [55]. The WAEC has been created based on existing trauma instruments, such as the War Exposure Scale (WES) [53] and the Life Events Checklist for DSM-5 (LEC-5) [56], and adapted to the Middle East refugee population. The resulting checklist consisted of ten items of war-related events and fifteen items of traumatic experience events and each item had two response options (yes, no).

#### PTSD

The validated Kurdish Kurmanji and Arab versions of the PTSD Checklist for DSM-5 (PCL-5) [53] were employed to assess PTSD symptoms. The PCL-5 is a self-report measurement and has been developed based on DSM-5 symptom criteria for PTSD. The scale consists of twenty self-report items which were categorized into four symptom clusters. For each item, there were five points with scores ranging from “Not at all” = 0 to “Extremely” = 4. Among the Kurdish and Arab population, PCL-5 had a high level of internal consistency and acceptable convergent validity; with the cut-off score of 23, the instrument obtained optimal balance of sensitivity and specificity [53]. The same high level of internal consistency (alpha = .89) was obtained with the Syrian refugees in the current study.

#### Depression

The depression subscale of the Kurdish Kurmanji and Arabic version of the Hopkins Symptom Checklist-15 (D-HSCL-15) [53] were used to examine depression symptoms. The D-HSCL-15 includes 15 items of depression symptoms. For each symptom, the severity is rated on a four-point scale ranging from “Not at all” = 1 to “Extremely” = 4. The D-HSCL-15 score was calculated by dividing sum scores of the items by the number of the items. A cut-off value of ≥1.75 was considered to be a case requiring treatment for major depressive disorder [56]. The scale is a common and widely used checklist to assess depression symptoms among refugees. It had also been translated to the spoken languages most commonly used at present among refugee populations, such as Farsi, Arabic etc. [57]. The depression subscale of the HSCL-25 was not specifically validated and calibrated for this population, but a high level of internal consistency (Alpha = .85) was found for the 15 items of the D-HSCL-15 utilized for the current sample.

### Statistical analysis

The Statistical Package for the Social Sciences (SPSS) program version 25 was used to carry out the statistical analyses. Descriptive analyses were executed to describe general characteristics of the participants, number of traumatic experiences, PTSD and depression symptom levels. Two-tailed independent sample t-tests were carried out to identify group differences in terms of PTSD and depression symptoms. Point-biserial correlations were used to identify relations between dichotomous variables and Spearman-rank correlations were used to test non-normally distributed variables. To check the normality of PTSD and depression scores, exploratory data analysis was used. To account for the fact that we used couples, the intra-cluster correlation coefficient (ICC) was calculated to estimate the statistical independence of the individuals within clusters. Based on the low values of ICC (PTSD sum score ICC = 0.0055 and depression sum score ICC = 0.034), we refrained from controlling the design effect and calculated standard multiple linear regressions to examine potential predictors of PTSD and depression.

## Results

### Traumatic experiences

**Fig. 1:**
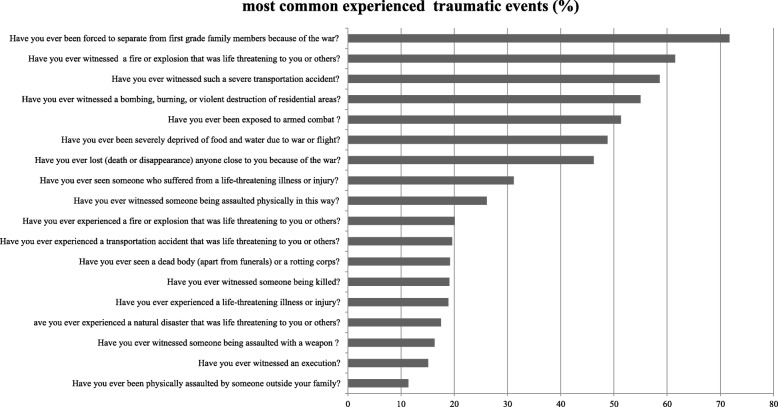
Most common experienced traumatic events (%)

Out of the 25 traumatic event types covered in the questionnaire, participants reported having experienced between 0 and 19 traumatic event types (*M* = 6.29, *SD* = 3.55). Approximately all of the participants (98.5%) experienced at least one traumatic event and the vast majority of them (86.3%) reported three or more traumatic event types during war or during their lifetime. Nearly three quarters (71.7%) of them had been separated from their family members during the course of the war and 51.3% reported that they had been exposed to armed combat (see Fig. 1). There was a significant difference (t (979) = 6.93, *p* < .001) between men (*M* = 7.06, *SD* = 3.77) and women (*M* = 5.52, *SD* = 3.13) in the number of the traumatic events they experienced.

### Mental health symptoms

The mean severity of PTSD symptoms was 26.44, *SD* = 15.3 (range = 0–58). Based on the recently validated cut-off score of 23 for the Kurdish and Arab version of the PCL-5 [53], 61.4% of the participants met DSM-5 symptom criteria for a diagnosis of probable PTSD. The difference in the level of PTSD symptoms between men (*M* = 25.49*, SD* = 16.38) and women (*M* = 27.38, *SD* = 14.1) just did not reach significance, *t* (984) = 1.94, *p* = .052. A significant difference was found in the level of PTSD symptoms between those who grew up in villages (*M* = 24.29*, SD =* 14.59) and those who grew up in cities (*M* = 27*, SD* = 15.57), (*t* (984) = 3.26, *p* = .001).

Regarding depression symptoms, the mean severity of depression symptoms was 29.36, *SD* = 8.52 (range = 15–57), using the cut-off score of 1.75, 59.4% of them were classified with probable depression. Women presented with significantly higher levels of depression (*M* = 30.53, *SD* = 8.94) than men (*M* = 28.19*, SD =* 7.9), *t* (984) = − 4.34, *p* < .001. There was no significant difference (*t* (984) = 1.49, *p* = .13) in the level of depression symptoms between those who grew up in villages (*M* = 28.81, *SD =* 8.45) and those who grew up in cities (*M* = 29.66, *SD* = 8.54).

### Predictors of mental health symptoms

Multiple linear regression analyses were carried out to examine potential predictors for PTSD and depression symptoms. For each of the outcome variables, we included the following variables as predictors: gender; age, years of formal education, number of children, years of life spent in camp, area of living while growing up, number of family members who were affected during war and displacement and number of traumatic events. Regarding PTSD symptoms, a significant regression equation was found *F* (8, 96) =25.85, *p* < .000), with an adjusted *R*^2^ of .169. Pertaining to depression symptoms, a significant model was found as well, *F* (8, 96) =21.61, *p* < .000), with adjusted *R*^2^ of .145. Results from these models showed that being female, older age, a more extended stay in camp, growing up in urban areas compared with the rural area and number of experienced traumatic events were significantly correlated with PTSD and depression. In addition, all of these factors (except older age for PTSD and growing up in urban area for depression) were among the significant predictors for PTSD and depression. The details of the analyses can be seen in Table 2.

**Table 2:** Predictors of PTSD and depression symptoms among the sample (*N* = 988)

Predictor	PTSD		Depression	
	Zero order correlation	Standardized ß- Coefficient	Zero order correlation	Standardized ß- Coefficient
Gender	.056*^b^	.171***	.099**^b^	.248***
Age	.104^***a^	.028	.133***^a^	.111*
Years of formal education	−.080* ^a^	−.039	−.068* ^a^	.015
Number of children	.131***	.088	.157*** ^a^	.082
Duration of stay in camp	.161*** ^a^	.165***	.122*** ^a^	.122***
Urban area of living while growing up	.084**^b^	.100**	.046^b^	.057
Number of family members who were affected during war and displacement	.235*** ^a^	.050	.168*** ^a^	.006
Number of traumatic event types	.308***^a^	.334***	.266***^a^	.290***

Note: *p ≤ .05, **p < .01, ***p < .001, ^a^ spearman correlation, ^b^ Point-biserial correlations

## Discussion

The current study demonstrated the psychological consequences of the Syrian conflict among Syrian Kurdish refugees residing in camps in the KRI. Almost all of the participants (98.5%) experienced at least one traumatic event and more than three quarters of them experienced three or more traumatic event types. This high level of traumatic events is similar to findings among a highly vulnerable sample of Yazidi women and girls in Iraq who survived enslavement and genocide in the context of the current war in the Middle East [55].

Around 60% of the participants met symptom criteria for probable PTSD. This prevalence rate is in line with prevalence rates found in other war-affected populations who had to flee from their home regions, such as internally displaced people in northern Uganda [58] or Kosovar refugees in the United States [30]. This rate of 60%, however, exceeded the prevalence rates of other populations such as Rwandese, Somali, Kosovar, and Iraqi refugees, which have ranged between 20 and 40% [32, 59, 60], as well as in other samples of Syrian refugees [27, 28, 36, 46]. For example, in the study of Ibrahim and Hassan, using the Harvard Trauma Questionnaire, the PTSD prevalence did not exceed 40% [27]. At the same time, using the Impact of Event Scale-Revised, the prevalence rate of probable PTSD in the current study is lower compared to a recent study of Syrian refugees residing in Turkey, which found that 83.4% of the sample were classified with probable PTSD [9].

The findings on depression point in a similar direction. In the current sample, 59.4% of the participants reported probable depression. This figure is in the range of findings of other global populations, such as IDPs in Kaduna, North West Nigeria [61]. The probable depression rate found in this study, however, exceeds numbers found in Kosovar and Somali refugees [62], Iraqi refugees located in Western countries [60], Syrian refugees residing in Turkey [9], and Syrian refugees in Lebanon [29].

Across studies, several factors might explain the heterogeneity of prevalence rates of PTSD and depression. It remains unclear to what extent the differences can be attributed to true differences of the populations, or to methodological factors such as sample selection and the selected instruments [22]. Due to the fact that the data in the present study are based on interviews applying instruments that had been validated for the population, we have reason to assume that our estimation comes close to the population parameters. There is only a few previous studies with larger sample sizes that used face-to-face interviews to diagnose PTSD [36], while other studies have relied on screening instruments such as the Impact of Event Scale-Revised and Beck Depression Inventory [9] with unknown precision in a Middle Eastern population.

In addition to limitations around methodological factors, the country of resettlement might influence the mental health of refugees. For instance, Syrian refugees who resided in Sweden reported lower levels of PTSD, psychiatric co-morbidity, and trauma characteristics relative to those residing in Turkey [46]. The context of the host country might include various post-migration factors that could be one of the sources of distress fostering the development of mental health problems among refugees [63]. Third, culture may play a significant role in determining mental health disorders as it has a significant contribution in the perception of traumatic events, where some events considered as traumatic within a specific culture may not be perceived as traumatic in other cultures [63]. Notwithstanding the heterogeneity of findings across studies, this study adds to the evidence that war-affected populations are characterized by excessive rates of PTSD and depression.

The dose-effect relationship between trauma exposure and mental disorders [64] was also confirmed in this study, since the number of experienced traumatic event types was correlated with PTSD and depression. Indeed, it was one of the largest predictors for these two mental problems. It is in line with the findings of Alpak and colleagues that showed a similar dose-effect relationship among Syrian refugees in Turkey [36] and various other studies worldwide [11, 23, 58, 65, 66].

This study revealed some gender differences in the sample. First of all, men reported having experienced more traumatic event types than women. This is consistent with results from previous studies [32, 58], including a recent study of Syrian Kurdish refugees in the KRI [27]. However, men and women did not show any significant differences in their levels of PTSD symptoms. This is similar to findings by Ibrahim and Hassan, who also did not find significant differences between men and women in PTSD symptoms among Syrian Kurdish refugees [27]. At the same time, women reported a higher level of depression than men in our sample. This finding is consistent with the results of most previous studies among internally displaced persons and refugees worldwide [58, 65, 67, 68]. The fact that, despite differences in trauma exposure, PTSD prevalence was similar argues for a higher vulnerability of women in this context. In a multivariate regression, gender (being female) was confirmed as predictor of PTSD, the result is exactly in line with previous studies [9, 32, 36, 69, 70]. This might be attributed to differences in the types of events experienced, since females are more likely to have experienced events that are more closely related to mental health consequences, in particular sexual violence and family violence [39, 71]. Further, as a result of war, women are often experiencing a number of major stressors like becoming widows or their husbands becoming disabled. These events increase the number of demands placed upon them, as they become responsible for providing for their families [9].

The present study revealed that growing up in urban areas is one of the risk factors for developing PTSD symptoms, even after controlling for trauma exposure. In general, mental health status is associated with living location, as mental disorders are more prevalent in the metropolitan cities, and mental diseases such as mood, anxiety, and psychotic disorders are generally more prevalent in cities than rural areas [47].

So far it is unclear if this effect reflects differences in population characteristics or vulnerability due to stress in urban areas. At the same time, it is necessary to consider that the Syrian war started as a conflict in urban areas where battles and bombings occurred in large cities, so systematic differences in war exposure that are not covered by the assessment of traumatic event types may account for this effect.

Our study confirms findings in the field about the risks to mental health that increase with more time spent in refugee camps [9]. As Acarturk and colleagues have shown, dissatisfaction with camp conditions was linked with experiences of depression. Further, the present study clearly demonstrates that life in the camps in the KRI themselves can be a stressful experience which should be considered as a major post-migration risk factor for developing mental illness among Syrian Kurdish refugees.

The strength points of the present study were that recently validated instruments for the targeted population were used to evaluate mental health problems and that all the interviews were conducted by local trained psychologists and social workers who were fluent in the Kurdish Kurmanji and Arabic languages. Moreover, the sample size was large (988 adults) which leads to increasing statistical power and providing more accurate results. The comprehensive assessment of psychopathologies provides links to expand on in future studies.

The major limitation of the current study is the limited representativeness of the sample. First of all, only couples were interviewed in this study, which leaves out possibly more vulnerable individuals like unmarried persons and widows. Previous researches have reported the association between marriage and mental health where married people are at low risk of psychiatric disorders compared with widowed and divorced adults [58, 72]. So, it is expected that the mental health of unmarried persons might be worse, but this still needs to be confirmed by conducting further research among this population. Second, there are more than eight camps for Syrian refugees (the vast majority of which are populated primarily by Kurds) in the KRI and more than half of the refugees are hosted within communities outside of camps, but the sample taken was from one camp only. As such, the results cannot be generalized to all Syrian Kurdish refugees. So, it will be valuable to conduct further research using the same instruments, among the same population but taking samples from different camps. Third, daily stressors as a factor that might be related to negative mental health were not evaluated in the current study. So, it is suggested for future research to assess daily stressors along with mental health symptoms, which might provide a clearer perspective. Then the results of these kinds of studies might be helpful during psychotherapy by focusing more on reducing daily stressors rather than just focusing on experienced traumatic events [1]. Fourth, Although we administered PCL-5 and D-HSCL by trained local clinical psychologists and social workers in the structured interview format rather than self-rating, still both of the instruments are self-reports, and they have a limited representation of DSM-V symptoms for PTSD and depression and also tend to show higher prevalence rates [73]. So, using clinical criteria among the same population might provide different prevalence rates of mental disorders and it will be an interesting research question for future research to compare validated self-report questionnaires with clinical criteria to estimate prevalence of mental disorders. Finally, caution is warranted in the interpretation of the prevalence rate identified in the present study. In contrast to the assessment of PTSD in this study, our depression instrument (D-HSCL) had not been specifically validated and calibrated for the population.

## Conclusion

The results of the present study showed that more than half of the sample in a refugee camp in Iraq are suffering from PTSD and depression. Further, the findings of the study suggest that PTSD and depression among Syrian refugees in the KRI might be serious mental health disorders, particularly among females, older individuals, those who grew up in large cities, and those who were exposed to a higher number of traumatic events. Thus, these results need to be considered during the psychological screening and intervention in this population. Since the present study is, to the best of our knowledge, one of the first to use recently validated instruments with a large sample of Syrian Kurdish refugees, the findings of the present study might be considered perspective first by international and local mental health organizations, governments, and the human rights agencies. These results may serve as a starting point for coming to a better understanding of the specific psychological demands of Syrian Kurdish refugees in order to facilitate more responsive and effective relief and rehabilitation. Next, it is suggested for all government, non-government, international, and national organizations and those who provide services in the camps, effective intervention, and better psychological support need more attention to the physical needs (food, water, camp conditions) first or at least along with psychological services. Finally, as a result of stigma and/or suffering from severe mental disorders, most of the refugees do not ask psychological services by their own. Therefore, regular visiting by psychologists and specialist health staff for the tents to ask those who are in need to use psychological services is recommended.

## Data Availability

The datasets generated during and/or analyzed during the present research are not publicly available to protect the anonymity of participants. Excerpts of the data are provided by the corresponding author upon reasonable request.

## Abbreviations

DHSCL: Depression section of the Hopkins Symptom Checklist
IDP: Internally Displaced Person
KRI: Kurdistan Region of Iraq
PCL-5: PTSD Checklist for DSM-5
PTSD: Posttraumatic Stress Disorder
WAEC: War and Adversity Exposure Checklist
